# Mapping, Bayesian Geostatistical Analysis and Spatial Prediction of Lymphatic Filariasis Prevalence in Africa

**DOI:** 10.1371/journal.pone.0071574

**Published:** 2013-08-12

**Authors:** Hannah Slater, Edwin Michael

**Affiliations:** 1 Department of Infectious Disease Epidemiology, Imperial College London, St. Mary's Campus, Norfolk Place, London, United Kingdom; 2 Department of Biological Sciences, University of Notre Dame, Notre Dame, Indiana, United States of America; Massey University, New Zealand

## Abstract

There is increasing interest to control or eradicate the major neglected tropical diseases. Accurate modelling of the geographic distributions of parasitic infections will be crucial to this endeavour. We used 664 community level infection prevalence data collated from the published literature in conjunction with eight environmental variables, altitude and population density, and a multivariate Bayesian generalized linear spatial model that allows explicit accounting for spatial autocorrelation and incorporation of uncertainty in input data and model parameters, to construct the first spatially-explicit map describing LF prevalence distribution in Africa. We also ran the best-fit model against predictions made by the HADCM3 and CCCMA climate models for 2050 to predict the likely distributions of LF under future climate and population changes. We show that LF prevalence is strongly influenced by spatial autocorrelation between locations but is only weakly associated with environmental covariates. Infection prevalence, however, is found to be related to variations in population density. All associations with key environmental/demographic variables appear to be complex and non-linear. LF prevalence is predicted to be highly heterogenous across Africa, with high prevalences (>20%) estimated to occur primarily along coastal West and East Africa, and lowest prevalences predicted for the central part of the continent. Error maps, however, indicate a need for further surveys to overcome problems with data scarcity in the latter and other regions. Analysis of future changes in prevalence indicates that population growth rather than climate change *per se* will represent the dominant factor in the predicted increase/decrease and spread of LF on the continent. We indicate that these results could play an important role in aiding the development of strategies that are best able to achieve the goals of parasite elimination locally and globally in a manner that may also account for the effects of future climate change on parasitic infection.

## Introduction

Recently, there has been increasing scientific interest in acquiring a better understanding of the spatial distributions of parasitic infections [1]–[13]. First, such work, by detailing the distribution and severity of diseases, is important for guiding the planning of control programmes [14]–[17]. Maps of prevalence or intensity of infection, for example, can enable a more precise stratification of disease risk faced by communities, which can in turn allow more reliable spatial planning of intervention efforts as well as identification of the worst affected areas for prioritizing these efforts [4], [8], [15]–[16], [18]. Second, mapping studies can, by combining spatial data on parasite prevalence with geographic information on biotic and abiotic ecological variables, be used to explore the underlying causes of infection risk, thus improving our understanding of the transmission ecology of parasitic infections [1], [19]–[22]. Finally, understanding the relationships between mapped disease prevalences and environmental/climactic factors is useful for examining how climate change may affect the long-term transmission and distribution of diseases [1].

Lymphatic filariasis (LF) is a major vector-borne parasitic disease endemic to the tropics, including sub-Saharan Africa. It is thought to represent the second largest health burden of any vector-borne disease worldwide [23], and this, together with improvements in drug-based treatments and diagnostic tools [24], has led to LF being considered as one of only six infectious diseases that could be "eradicable" or "potentially eradicable" at the global level [25]. This conclusion followed by adoption of Resolution WHA50.29 by the World Health Assembly in 1997 calling for the elimination of LF as a public health problem, has resulted in the rapid implementation of large-scale national mass drug administration programmes in all endemic regions of the world [26]–[27]. This progress has led to impressive reductions in infection in treated communities, but has also made gaining a better understanding of the geographic distribution of LF prevalence a key requirement for more effectively guiding successful elimination activities across endemic regions.

Previous attempts to map LF in Africa have estimated infection distribution or prevalence across specific regions or countries by mapping of infection sites either as points or as ranges interpolated between such points [22], [28]–[32], quantifying spatial patterns using geostatistical approaches [3], [33] or smoothing aggregated prevalence data across areas to produce national level maps [34]–[37]. By contrast, Lindsay and Thomas [38] used logistic regression with environmental covariates and LF presense/absence data to predict the probability of LF presence across Africa, which was used to improve estimations of the number of people living in at-risk areas.

The importance of explicitly accounting for spatial effects when attempting to map infection data has been emphasized recently [6], [39]–[40], and a variety of methods for dealing with spatially correlated data has been suggested, within both the frequentist [41] and Bayesian frameworks [42]–[44]. Generalised linear models with spatially correlated random effects are commonly used in this context [39], and such generalized linear spatial models (GLSMs) have been used to map a wide range of phenomena, including germinating seeds [45], root rot [46] and bird populations [47]. They have also been increasingly used to model and predict disease prevalences, including schistosomiasis [4], [7], [48], pseudorabies virus [49], trypanosomiasis [9], and malaria [18], [50]. Despite this trend, no study thus far has modelled the prevalence of LF across an endemic region by addressing both environmental covariates and spatial effects together. This is in spite of the fact that 1) the spatial distribution of LF has been shown to be highly non-homogenous between and within countries [33]–[36], [51]; and 2) both intrinsic (aggregation and dispersal) and extrinsic environmental factors affecting the demographic rates of the vector and the developmental rates of the parasite within the vector [38], [52]–[53] are likely to vary spatially [3].

Here, we present a first attempt to develop a smooth map of LF infection prevalence across Africa using a Bayesian GLSM fitted to published community-level infection data and spatially varying demographic and environmental covariates expected to underlie the transmission of this parasitic infection in endemic regions. Recently, this analysis method has received increasing focus as a more rigorous means to obtain estimates of parameters for large classes of complicated models, including, as in the present case, for complex spatial modelling problems that require explicit accounting of spatial autocorrelation while incorporating uncertainty in input infection data and model parameters [4]–[7], [9]. The resulting prevalence map is used to investigate the geographical limits and levels of infection prevalence, the size of the population likely to be infected, and the environmental ecology of LF infection on the continent. It is predicted that future changes in climate may have an impact on the burdens of infectious diseases [54], particularly vector-borne diseases [55]–[56], and thus a second major aim of this study was also to undertake a first investigation of the potential impact global warming could have on future LF spatial distribution, prevalence and burden on the continent of Africa.

## Methods

### LF Prevalence Data

LF prevalence data for model building were collated from published community surveys conducted across Africa from 1940 to 2009, using the online and manual search procedures described previously [51]. Studies were selected if the surveys described the number of people surveyed, the number positive for microfilaraemia (mf), and were conducted at a specific community site. We found a total of 664 community-specific datapoints providing this information and these were used in the present analysis (see details of selected studies in **Table S1** in Information S1). Since field surveys employed different blood sampling volumes for detection of mf, all prevalence values were standardized to reflect sampling of 1 ml blood volumes using a transformation factor of 1.95 and 1.15 respectively for values originally estimated using 20 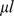
or 100 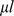
blood volumes [57]. Geo-coordinates for mapping of each chosen datapoint were either referenced from coordinates given in the literature or by using Google Earth. The corresponding author may be contacted for access to these data.

### Environmental Layers

A number of environmental and climatic variables, essentially related to temperature and percipitation, affect the development and survival of the *Wuchereria bancrofti* parasite and its transmitting mosquito vectors [38], [52]–[53]. Here, we began by choosing nine environmental data layers for analyses (**Table 1**), based on both their availability and biological plausibility in reflecting these external drivers of LF transmission. In addition, a spatial data layer describing the population density of each site was also included in the analysis to take explicit account of the role that host density plays in the transmission of vector-borne diseases, including LF [12], [58]–[59]. The climate and altitude layers were downloaded from http://www.worldclim.org on a spatial resolution of 10 km×10 km. The ground vegetation cover index (NDVI) and the population density layer for Africa were downloaded from http://edit.csic.es/GISdownloads.html and http://sedac.ciesin.columbia.edu/gpw/global.jsp respectively. Worldclim layers are smooth maps of climatic variables created using interpolated weather station data [60]. The NDVI layer was created using satellite sensor data (from NASA’s NOAA AVHRR sensor) and the population layer was created using data from, amongst others, the Socioeconomic Data and Applications Center (SEDAC) at Columbia University. The two sources of data were on slightly different scales, and so were resampled using ArcGIS to give all the layers the same grid size. This resulted in a scale of around 12 km×12 km for all layers used in this study. The WorldClim layers used for developing the model were monthly and annual means of temperature and percipitation estimated for Africa for the period 1950–2000. The mean NDVI data are based on monthly values obtained over a 18-year period from 1982 to 2000. All other covariate data layers reflected data assembled for the year 2000. Covariate values were extracted from the data grids for each geographic location where a LF survey result was available.

**Table 1:** Details of variables used in the Bayesian geostatistical analysis.

Environmental Variable	Source	Details
Altitude	http://www.worldclim.org	–
NDVI	http://edit.csic.es/GISdownloads.html	Mean of average monthly mean temperature across all 12 months
Annual mean temperature	http://www.worldclim.org	Mean of average monthly mean temperature across all 12 months
Mean maximum temperature	http://www.worldclim.org	Mean of average monthly maximum temperature across all 12 months
Mean temperature in warmest month	http://www.worldclim.org	Maximum of the 12 average monthly maximum temperature layers
Mean minimum temperature	http://www.worldclim.org	Mean of average monthly minimum temperature across all 12 months
Mean temperature in coldest month	http://www.worldclim.org	Minimum of the average monthly minimum temperature layers
Mean annual precipitation	http://www.worldclim.org	Mean of average monthly precipitation across all 12 months
Precipitation in wettest month	http://www.worldclim.org	Maximum of the 12 average monthly precipitation layers
Population density	http://sedac.ciesin.columbia.edu/gpw/global.jsp	–

### Multivariate Bayesian Generalised Linear Spatial Model

We used a Bayesian GLSM to spatially model the community-based LF infection data in response to the multiple environmental/demographic covariates derived at each site. If we assume that the number of individuals found positive for LF at location 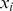 is 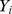 out of the total number of people tested, 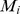, then 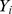 is a binomial random variable, 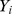 ∼ *Bin(M_i_, p_i_)*, where *p_i_* is the proportion of individuals infected at each location. A multivariate GLSM can be used to fit this model to observed data with the predictor variables entered as fixed effects and the spatial structure in the residuals modelled by inclusion of a location-specific random effect, 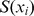 [61]. The model is denoted 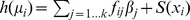 where 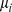 is the expected value of infection prevalence at location 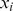 conditional on a random spatial process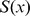, 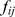 represents the value of the 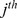 environmental variable at the 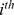 location and 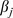 is the coefficent of the 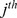 variable. The link function is denoted by 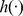; for binomial data we use the logit function 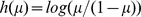. 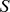 is modelled as a stationary Gaussian process with 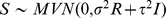 where *I* is the identity matrix, and 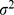 and 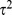 represent the spatial variance and the nugget effect (which accounts for the additional non-spatial variation in the data) respectively. 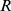 is an 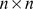 correlation matrix with 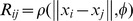 with an exponential spatial correlation function dependent on the parameter 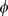.

### Model Estimation

Given our Bayesian modelling framework and initial uncertainty regarding parameter values, we assigned vague normal prior distributions with mean 0 and variance 5 for all the variable coefficients and the intercept. These values were selected by running a standard, *ie.* non-spatial Generalized Linear Model (GLM), examining the magnitudes of the regression coefficients and intercept, and selecting prior distributions for each parameter with a wide variability centred near the mean value. The parameter 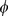, which controls the rate of exponential decay of the spatial correlation process, is assigned a reciprocal prior distribution between 4 and 6. We assigned 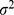 a uniform prior and we assume 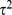 to be proportional to 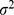, with 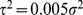, informed by an initial variogram analysis of the data. We modelled the spatial correlation between sites with a Matern function with 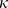 = 0.5, which equates to the use of an exponential function for describing spatial correlation in the data.

### Implementation and Convergence Diagnostics

The model was implemented using *geoRglm* [62], a software package based on the R statistical system. Model fitting was done by running 150,000 Markov Chain Monte Carlo (MCMC) iterations after a burn-in of 300,000 runs, and thereafter storing every 250^th^ element. Stability of model parameters was assessed by examining both the within and between chain convergence of the stored MCMC runs. Within chain convergence was analysed using two methods: 1) visual checks using trace plots, and 2) examining the MCMC error as a percentage of the standard deviation, with a MCMC error of less than 5% of the standard deviation taken to indicate satisfactory convergence [63]. The between chain convergence was evaluated by running five identical models and comparing both the obtained Deviance Information Criterion (DIC) values and using the the Gelman-Rubin (GR) diagnostic [64], which assesses convergence by running parallel chains from different starting values and determining if all chains converge to the same posterior distribution. The mean values of each regression parameter and the 95% and 80% credible intervals were calculated.

### Variable Selection and Model Development

We use a similar procedure as outlined in Austin and Tu [65] and Craig et al. [66] to select a set of predictive variables that are uncorrelated for developing the multivariate Bayesian GLSM. The rational behind this approach is that often due to convergence and mixing problems with the MCMC sampling approach when including all of the spatial covariates (as was the case for the present analysis), the most practical route is to reduce the list of potential correlated explanatory variables using non-spatial selection methods, before moving to a spatial context [9], [66]. The approach comprised the following steps.

Step 1– We performed a non-spatial univariate logistic regression analysis relating the observed infection prevalences to each of the covariates, and recorded the Akaike Information Criterion (AIC) of each fitted model [67]. AIC is defined by: −2*L*(β)+2*k*. Here β = {β_0_, β_j_} are the regression coefficients, *L* is the maximum value of the likelihood function for the model and *k* is the number of parameters included in the model, which indicates that AIC penalizes for the addition of parameters.

Step 2– We reduced multicollinearity and confounding effects arising from correlated variables by identifying all pairs of variables with a Spearman’s rank >0.7 and eliminating the variable in each pair from an environmental theme (eg. set of temperature or precipitation variables) giving the highest AIC value as derived in step 1. We also examined scatter plots of each pair of variables for the existence of any non-linear correlations.

Step 3– Next, we determined the functional form of the variables by fitting two different functional forms, a linear versus quadratic form, to the data, and calculated the AIC of each model. The functional form with the lowest AIC was selected.

Step 4 - The GLSM described above was then run with the variables selected from step 2 and the functional forms identified in step 3 using the *binom.krige.bayes* function in the geoRglm package. We performed a manual stepwise variable selection procedure whereby we removed one variable at a time, reran the model and assessed model performance using DIC [68]. If model performance improved, the variable was retained and if not, it was eliminated. This was repeated for all variables and continued until all remaining variables contributed to model performance.

### Model Validation

We assessed the predictive ability of the final model by removing 100 data points and then fitting the Bayesian spatial model to the remaining data. The fitted model was used to make predictions over the removed data locations, and model accuracy was assessed by comparing the model prediction and observed prevalence for each location. The predicted LF infection prevalence was classified as correctly predicted when the observed prevalence for a location was within the 95% Bayesian credible interval (BCI) or within the 75%, 50%, 25% and 5% BCIs resulting from the predictive posterior distribution of that location [7], [69].

### Spatial Predictions

For mapping, we predicted infection prevalences using the final selected model at selected grid locations covering the whole of Africa. The predicted values were posterior means realised as part of the MCMC simulations from the posterior predictive distribution [61]. Approximate standard errors were obtained by dividing the 95% credible intervals by 4. Due to computational limitations we resampled the grid to create a coarser grid with cells of around 60 km×60 km using ArcGIS. This reduced the number of prediction sites from 500,000 to just under 20,000.

### Estimating the Population with LF

The number of people infected with LF in Africa was estimated by overlaying the estimated prevalence map with the population map for the year 2000. We chose to use the 2000 population data for this calculation not only to conform with the climate data, but also to estimate and present the baseline infection burden of LF in Africa prior to the initiation of large scale control programs in endemic countries, which began in ernest only after the year 2000 on that continent. The number of infected people was calculated by multiplying estimated prevalence by population on a cell-by-cell basis. The mean prevalence and the total number of people with LF in each country were then estimated using the *zonal* statistics function available in the spatial analyst package in ArcGIS (ArcMap 9.3).

### Future LF Predictions

In order to estimate the impact of future climate change and population growth on the spatial distribution of LF, we applied the parameter estimations from the current model fitted to baseline data to the climate and population data from 2050, based on the simplifying initial assumption that the biological relationships governing disease transmission would remain largely unchanged over the two estimation periods. The results were used to both examine how prevalence might change from pre-intervention levels in currently defined LF endemic areas as well as whether new areas might become suitable for LF transmission in the future. We used climate predictions from two climate models – the Hadley Centre global climate model HADCM3 and the Canadian Centre for Climate Modelling and Analysis model CCCMA - under two IPCC climate scenarios – A2a and B2a [70]. A2a is a scenario assuming large disparities between regions and high population growth and energy use. B2a aims to capture a less disparate world with efforts focused towards social equity; this scenario assumes lower population and economic growth than A2a. To account for differences in population growth between the two climate scenarios we multiplied the 2000 population data by the country specific UN medium variant population growth rate predictions for the B2a scenario and by the high variant growth rate predictions for the A2a scenario (http://esa.un.org/unpd/wpp/unpp/panel_population.htm).

## Results

### Sample Site Locations and Observed LF Prevalence

**Figure 1:**
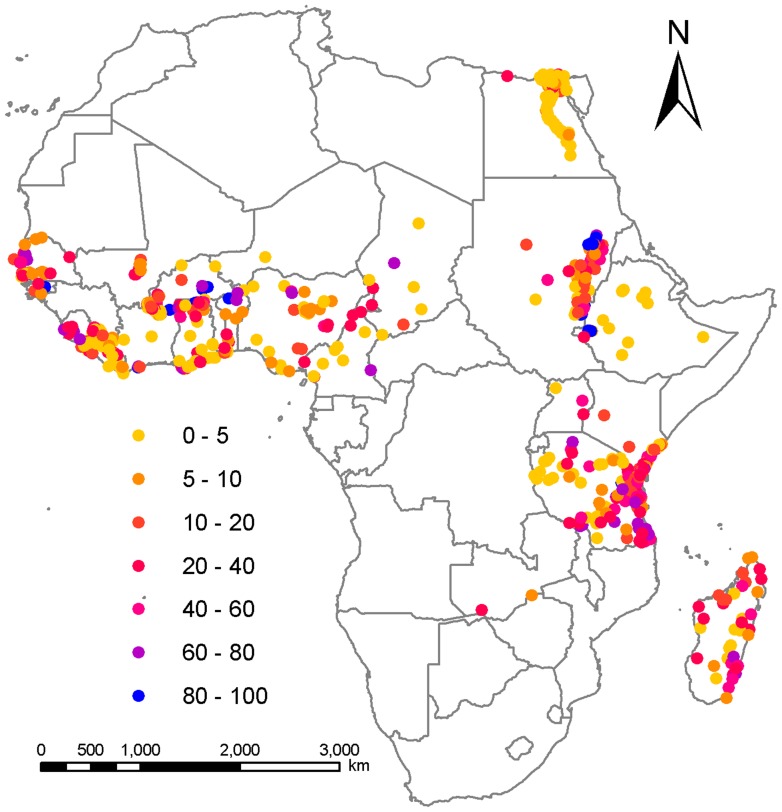
Locations and prevalence of LF infection for each study survey used in the present analysis. Points are coloured in relation to the percentage of survey population with mf in their blood.

The locations of the survey sites used in this study with their observed LF mf prevalence are shown in **Figure 1**. The depicted map shows that most LF community surveys carried out in Africa have occured along the western and eastern regions of the continent, across Sudan and Ethiopia, along the river nile in Eygpt, and on the coast of Madagascar. By contrast, very little information on infection status and prevalence are available from communities in the central regions (**Figure 1**). The observed community infection prevalences are also highly spatially heterogeneous, with sites exhibiting high prevalences occuring generally towards the central western and eastern regions of the continent, along a central gradient in the north eastern region and along the coast of Madagascar. By contrast, the surveyed community infection prevalences appear to be lower towards the southern parts of the western region, the central portion of the East-West endemic band and along the river Nile in the northern reaches of the continent (**Figure 1**).

### Variable Selection and Univariate Analysis

Following steps 1–2 in the methods, we identified four pairs of variables with spearman’s rank >0.7. From each correlated pair, we selected the variable with the lowest AIC from step 1 for inclusion as a predictor in the model. Non-linear correlations between variables were assessed visually, but none were identified (see **Figure S1** in Information S1). This left five out of the original 10 variables (**Table 1**) for inclusion in model development, *viz.* altitude, NDVI, population density, mean precipitation in the wettest month and mean annual temperature.

**Figure 2:**
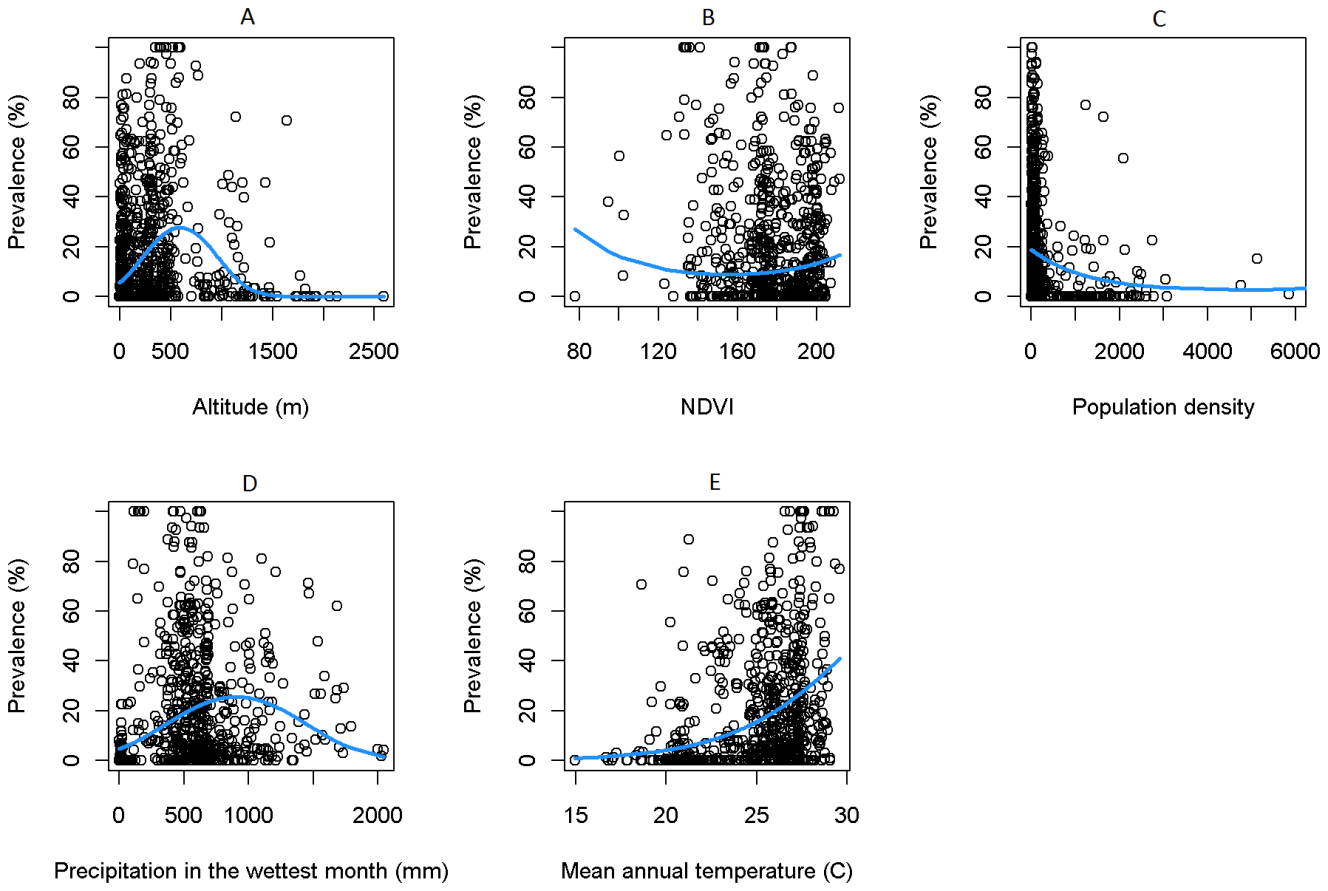
Scatter plots of each ecological/environmental variable against LF prevalence. The blue lines denoted the fitted quadratic functional forms for each variable.

**Figure 2** depicts the univariate relationship between LF prevalence and each of the selected variables or covariates. The lines show the mean fits of quadratic logistic regression models for each variable. Comparison of these fits with simple linear logistic relationships indicated that in every case the quadratic form provide a better fit to the data (not shown), suggesting that the relationship of each variable with LF prevalence was highly and significantly non-linear. Given this result, we assigned quadratic functional forms to all the six variables selected as predictors in this study.

### Multivarate Bayesian GLSM Model

The results of fitting the Bayesian GLSM model to the LF prevalence data are shown in **Table 2**. The DIC values given in the table show that the full Bayesian GLSM (*ie.* with covariates and spatial component) gave the best, parsimonious, fit to the data in comparison with models containing only a spatial component and only covariates. The model with no spatial term included is the worst model, with a large DIC value of 71,246 (**Table 2**), clearly indicating the need for accounting for spatial correlation in the data. The non-spatial multivariate analysis shows that all covariates are associated with the risk of mf, but implementation of the full multivariate GLSM indicated a loss of importance for all the environmental and climatic variables. The spatial term appeared important even when 95% credible intervals are used for the full GLSM but in the case of covariates, only population density is shown to be required in the model when both the 95% and the lower 75% credible intervals were used for judging variable importance (**Table 2**). However, running a reduced spatial model containing only this variable produced an increase in the DIC value (to 130), and also did not improve model performance in correcting predicting observed LF prevalence at validation locations (see below) compared to the full model. This suggests that considering the full set of covariates as selected in the full GLSM is required for predicting LF mf prevalence distribution across Africa.

**Table 2:** Model parameters and the 95% and 75% credible intervals for the full model, and parameter values and 95% CIs for the model with no covariates and the model with no spatial term. Significant covariates in each model are highlighted in bold.

	Full model			No covariates		Population density model		No spatial term	
Coefficient	Mean	95% CI	75% CI	Mean	95% CI	Mean	95% CI	Mean	95% CI
*β*_0_ – intercept	−16.06	(−37.035,8.601)	(−29.160,−2.504)	−3.421	**(−6.988,** **−0.011)**	−2.775	(−6.585,1.457)	1.233	**(0.943, 1.524)**
*β*_1_ – altitude	−0.102	(−0.573,0.318)	(−0.331,0.146)					0.120	**(0.107, 0.131)**
*β*_2_ – altitude^2^	−0.002	(−0.022,0.017)	(−0.014,0.010)					−0.017	**(−0.018, −0.016)**
*β*_3_ – NDVI	5.188	(−4.245,14.301)	(−0.200,10.894)					0.304	**(0.023, 0.580)**
*β*_4_ – NDVI^2^	−1.673	(−4.600,1.419)	(−3.548,0.092)					−0.124	**(−0.209, −0.038)**
*β*_5_ – population density	−0.240	(−0.63,0.114)	**(−0.463,** **−0.040)**			−0.262	(−0.604,0.112)	−0.277	**(−0.298, −0.256)**
*β*_6_ – population density^2^	0.037	**(0.003,** **0.075)**	**(0.017,** **0.059)**			0.038	**(0.006,** **0.073)**	0.023	**(0.021, 0.025)**
*β*_7_ – prec in wet month	0.189	(−0.385,0.733)	(−0.139,0.540)					0.266	**(0.253, 0.279)**
*β*_8_ – prec in wet month^2^	−0.009	(−0.033,0.015)	(−0.026,0.006)					−0.012	**(−0.013, −0.011)**
*β*_9_ – mean annual temp	0.698	(−1.293,2.444)	(−0.447,1.759)					−0.375	**(−0.408, −0.342)**
*β*_10_ – mean annual temp^2^	−0.013	(−0.050,0.029)	(−0.036,0.012)					0.009	**(0.008, 0.010)**
*σ* ^2^ – spatial variance parameter	61.686	**(55.229,** **68.277)**	**(57.655,** **65.839)**	54.676	**(48.171,** **62.542)**	62.763	**(56.217,** **69.247)**		
*φ* – spatial decay parameter	5.507	**(5.488,** **5.522)**	**(5.498,** **5.516)**	4.875	**(4.567,** **4.995)**	5.485	**(5.424,** **5.545)**		
DIC	118.8			1100.7		130.3		71246	

Both trace plots and the Geldman-Rubin diagnostic indicated good convergence for all the β and σ^2^ parameters of the model (see **Figure S2** and **Table S2** in Information S1). However, the results indicate that the decay parameter for spatial correlation (φ) may not have converged adequately, suggesting that the derived value for this parameter requires to be treated with caution. The posterior distributions for all parameters were normally distributed.

**Figure 3:**
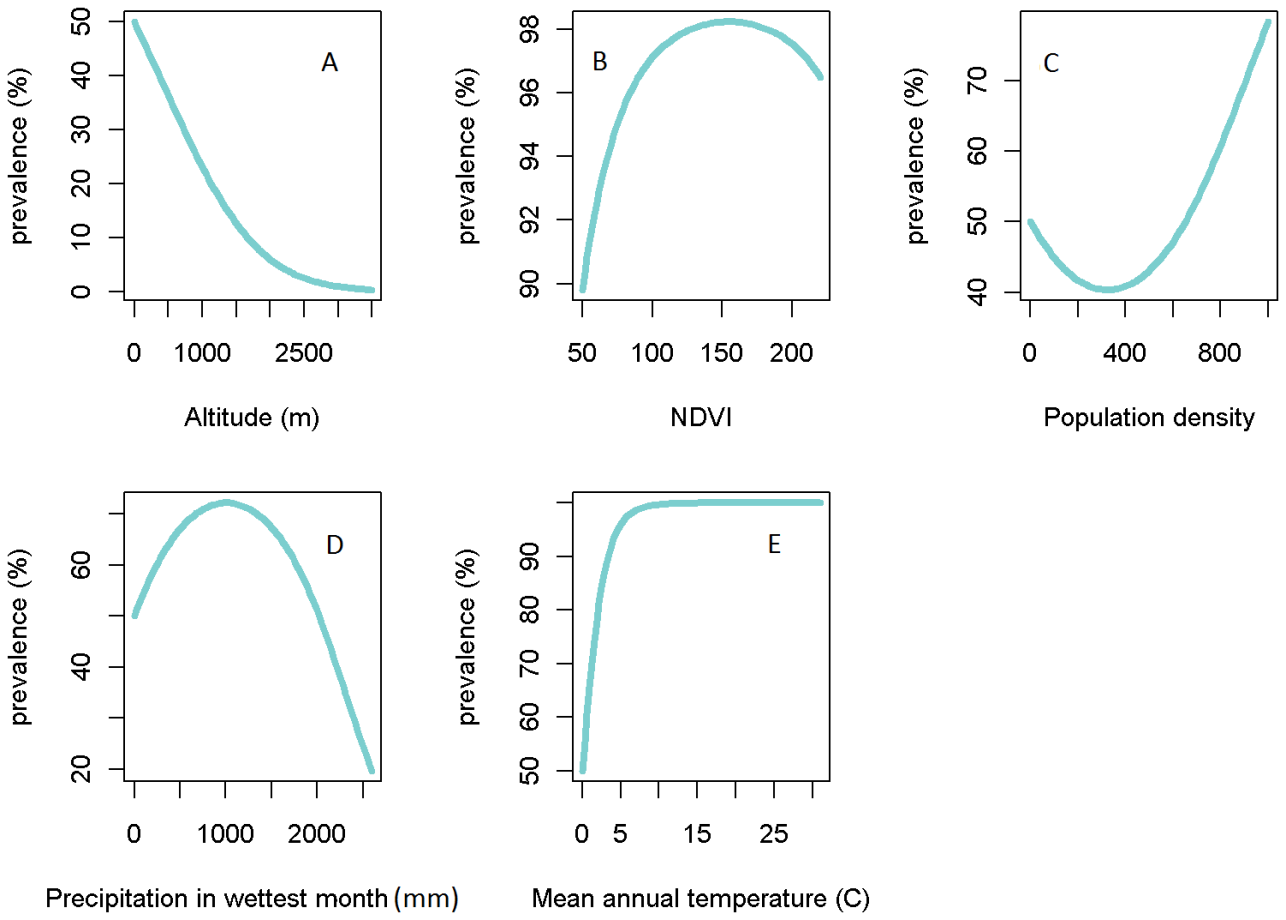
Functional forms for each ecological/environmental plotted using the coeficients estimated using the final multivariate bayesian spatial model. Lines show the marginal relationships estimated between each environmental variable and the prevalence of LF.

The functional forms of all the variables estimated using the full multivariate GLSM are portrayed in **Figure 3**. Interestingly, the forms for each variable uncovered by the multivariate model, except for precipitation in the wettest month, showed important differences with those detected for these variables using the univariate logistic regressions shown in **Figure 2**. Thus, compared to the corresponding univariate forms, LF prevalence is quantified to show a negative association with altitude, a generally positively increasing association with NDVI as well as with population density, and a monotonically increasing relationship with mean annual temperature (**Figure 3**). Given that the multivariate system facilitates parameterization of variables by taking account of the concurrent effects of other variables on the dependent response, and that the quantified functional responses are ecologically plausibile (see Discussion), we used the functions quantified by the full multivariate GLSM in all subsequent analyses in this paper.

### Model Validation

**Table 3** shows the mean number of test locations (from a sample of 100 randonly selected locations) with observed LF prevalences that fell into each of the selected BCIs of the posterior predictive distribution. The results show that the Bayesian spatial model with covariates performed better in predicting the number of test locations than the model without covariates across all the BCIs.

**Table 3:** Precentage of study locations with LF prevalence falling in the 5%, 25%, 50%, 75% and 95% Bayesian credible intervals for the geostatistical models without and with covariates.

	Credible Interval				
	5%	25%	50%	75%	95%
LF – with covariates	5.0±1.1	26.8±3.9	49.2±6.6	66.4±4.2	76.6±3.2
LF – without covariates	4.0±0.9	22.6±3.1	41.6±4.5	57.0±1.6	69.2±2.0

### Spatial Predictions

**Figure 4:**
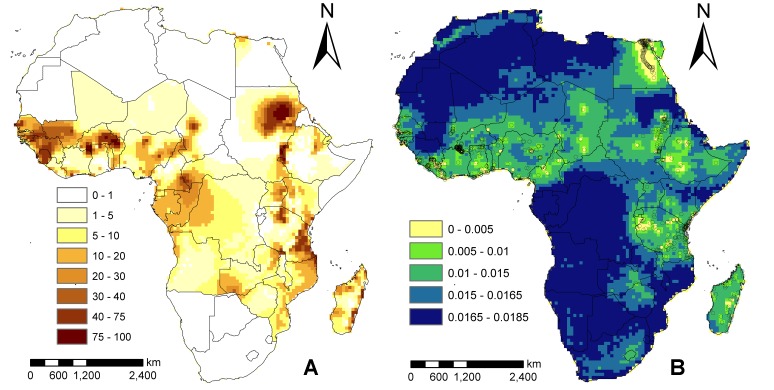
Predictions of the final Bayesian spatial model. (A) Estimates of LF prevalence in Africa. Known filariasis free territories have been masked out (Western Sahara, Mauritania, Morocco, Libya, Algeria, Tunisia, Eritrea, Somalia, Botswana, Namibia, South Africa, Lesotho and Swaziland – information from http://www.taskforce.org/). Values plotted are the mean of the posterior distribution from the multivariate Bayesian GLSM. (B) Map of the estimated standard errors with points showing the location of the LF prevalence data.

The risk map of predicted LF prevalence created using the full Bayesian GLSM is depicted in **Figure 4**. According to the predictions of mean prevalence (**Figure 4a**), areas of high LF prevalence (>20%) are estimated to be in coastal and north West Africa, around the Sudanese region, and along the East African coast. Medium-prevalence areas (10–20%) are predicted to occur in the west, central west, and along the south eastern borders of the LF endemicity zone. Interestingly, regions of some countries for which we had no or sparse data were found to be LF endemic, with some areas in central and southern Africa estimated to have prevalences of up to as high as 10%. However, examination of the estimation error map in Figure 4b shows that the areas with the lowest standard errors and hence uncertainty are as expected those where we have the most sample data while unsampled areas are associated with very high standard errors. This suggests that the mean prevalence predictions for the unsampled areas need to be viewed with caution. It also highlights how mapping of error estimates from running Bayesian GLSMs can be used to conduct secondary sampling of sites to fill in gaps in the currently available spatial data on community infection prevalences in an endemic region. The present results thus suggest that more community surveys of LF infection are urgently required in countries across the northern and southern most edges of the presently endemic LF zone in Africa, and in the central and south west parts of the continent (**Figure 4b**) if we are to develop more reliable maps of LF prevalence for this continent.

### Number of People Infected with LF

The estimated number of people with LF and the mean prevalence in each country for 2000 prior to the initiation of large-scale national-level mass drug administration are shown in **Table 4**. The results indicate that the baseline mean LF prevalence for the whole of Africa was around 7.85%, and that some 61.55 million people were infected with *W. Bancrofti* at the start of the current control campaign. Infection prevalence and the number of people infected, however, varied a great deal between endemic countries, with prevalence ranging from as low as 0.3% in Burundi to as high as 38% in Sierra Leone (**Table 4**). We predict that prior to 2000, Nigeria had the most people infected (11.01m), with significant infected populations (up to some 27.11 million or 44.05% of the total infected African population) also quantified for Burkina Faso, Egypt, Senegal, Sudan, Tanzania, Uganda and Zaire (**Table 4**).

**Table 4:** Estimated number of people with LF infection in Africa based on the full GLSM.

Country Name	2000	2050 A2a scenario	2050 B2a scenario
Algeria	0m (0%)	1.09m (2.1%)	0.91m (2%)
Angola	0.80m (6.1%)	3.63m (8.4%)	3.2m (8.3%)
Benin	0.90m (14.4%)	3.97m (18.3%)	3.45m (18%)
Botswana	0m (0%)	0.17m (5.8%)	0.14m (5.6%)
Burkina Faso	3.05m (26.5%)	11.71m (26%)	10.61m (26.4%)
Burundi	0.02m (0.3%)	0.34m (2%)	0.23m (1.6%)
Cameroon	1.52m (10.2%)	3.66m (9.4%)	3.21m (9.4%)
Central African Republic	0.40m (10.9%)	1.06m (11.8%)	0.92m (11.7%)
Chad	0.68m (8.6%)	2.27m (7.8%)	2.14m (8.2%)
Congo	0.38m (12.3%)	1.1m (13.1%)	0.96m (12.9%)
Djibouti	0.03m (4.7%)	0.06m (4.5%)	0.05m (4.5%)
Egypt	2.61m (3.9%)	11.76m (8.5%)	9.87m (8.2%)
Equatorial Guinea	0.04m (8.9%)	0.07m (5.5%)	0.05m (4.9%)
Eritrea	0m (0%)	0.71m (5.9%)	0.63m (5.9%)
Ethiopia	1.45m (2.3%)	5.22m (2.8%)	4.52m (2.7%)
Gabon	0.18m (14.9%)	0.2m (7%)	0.17m (6.8%)
Gambia, The	0.16m (14.2%)	0.22m (6.2%)	0.18m (5.8%)
Ghana	1.14m (6%)	4.28m (8.5%)	3.84m (8.7%)
Guinea	1.86m (23%)	4.67m (18%)	4.03m (17.5%)
Guinea-Bissau	0.28m (25.7%)	0.64m (18.8%)	0.49m (16.2%)
Ivory Coast	1.14m (7.2%)	3.07m (7.1%)	2.85m (7.5%)
Kenya	2.25m (7.4%)	8.53m (9.4%)	7.1m (8.9%)
Lesotho	0m (0%)	0.01m (0.2%)	0m (0.2%)
Liberia	0.29m (9.9%)	0.32m (3.2%)	0.26m (2.9%)
Libya	0m (0%)	0.81m (7.8%)	0.62m (6.7%)
Madagascar	1.73m (11%)	4.75m (9.6%)	4.36m (10.1%)
Malawi	0.59m (5.3%)	2.69m (6.9%)	2.18m (6.3%)
Mali	1.99m (17.6%)	6.62m (19.2%)	5.89m (19.2%)
Mauritania	0m (0%)	0.55m (7.9%)	0.52m (8.5%)
Morocco	0m (0%)	1.16m (2.4%)	0.95m (2.3%)
Mozambique	1.84m (10.2%)	6.02m (12.5%)	5.37m (12.8%)
Namibia	0m (0%)	0.2m (5.3%)	0.17m (5.1%)
Niger	0.29m (2.7%)	2.41m (3.8%)	2.2m (3.9%)
Nigeria	11.01m (9.7%)	31.79m (11%)	27.86m (10.8%)
Rwanda	0.04m (0.5%)	0.85m (3.6%)	0.61m (2.9%)
Senegal	2.85m (32.1%)	5.35m (20.5%)	4.73m (20.5%)
Sierra Leone	1.62m (37.6%)	3.94m (27.7%)	3.48m (27.7%)
Somalia	0m (0%)	1.11m (3.8%)	0.98m (3.7%)
South Africa	0m (0%)	1.83m (2.9%)	1.45m (2.7%)
Sudan	7.04m (22.7%)	18.09m (23.7%)	16.11m (24%)
Swaziland	0m (0%)	0.09m (5.4%)	0.08m (5.2%)
Tanzania, United Republic	5.40m (15.6%)	21.05m (17.2%)	18.45m (17%)
Togo	0.72m (16.2%)	0.85m (6.8%)	0.77m (7%)
Tunisia	0m (0%)	0.6m (4.6%)	0.44m (3.9%)
Uganda	2.97m (12.8%)	15.27m (15.6%)	13.07m (15%)
Western Sahara	0m (0%)	0.04m (3.8%)	0.04m (3.7%)
Zaire	3.19m (6.3%)	12.07m (7.3%)	10.64m (7.3%)
Zambia	0.76m (7.3%)	2.81m (8.6%)	2.4m (8.3%)
Zimbabwe	0.33m (2.6%)	1.09m (4.2%)	0.89m (4%)
Total population infected	61.55m (7.86%)	210.82m (9.85%)	184.03m (9.75%)

### Future Changes in LF Spatial Distribution

**Figure 5:**
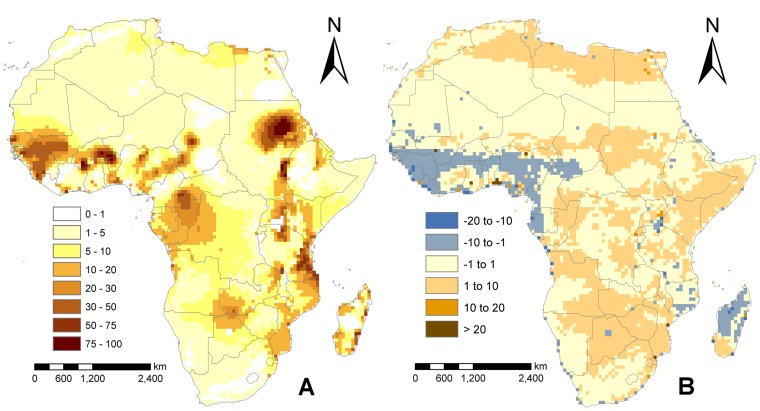
A) Predicted LF prevalence in 2050 using the CCMA model and climate scenario A2a, and B) percentage change in LF prevalence between 2000 and 2050. Areas in grey and blue are where prevalence is predicted to decrease, while areas in orange and brown are where it is predicted to increase.

We produced predictive LF prevalence smooth maps for Africa using the the climate and population density data for 2050 under each of the four climate change scenarios outlined in the Methods. The results were almost identical for both models and scenarios, with the mean prevalence across Africa estimated to be between 8.35 to 9.85% for all scenarios, and thus we illustrate results from the CCCMA model only in Figure 5 and Table 4. **Figure 5a** depicts the predicted prevalence in 2050, while **Figure 5b** shows the absolute change in estimated prevalences between 2000 and 2050. In constructing the latter result, we simply considered currently LF free countries to have 0% infection prevalence and used this fixed baseline value to calculate the absolute precentage change between the present and the predicted 2050 prevalence in these areas. The absolute change results sumarized in **Figure 5b** reveal firstly that the potential transmission areas of LF in Africa will significantly expand by 2050; in particular, both the northern and southern extremes of the continent will become appreciably endemic, with some currently low endemic areas in these regions predicted to reach infection prevalences >5% (**Table 4**). The second feature of the results shown in **Figure 5b** is that LF prevalence is also likely to change within the currently endemic countries/regions, significantly increasing in some areas while decreasing drastically in others (**Figure 5b** and **Table 4** - where countries with significant decreasing LF prevalence are highlighted in blue), although in the majority of these countries the change is predicted to be positive (**Table 4**). The estimated population with LF infection in 2050 based on the A2a scenario is predicted to be around 211m and 208m using the two climate models, while based on the B2a scenario, the estimated infected populations are calculated to be 184m and 179m respectively. However, as the underlying at-risk or exposed population in Africa will increase significantly by 2050 (due to both rapid population growth and spread of infection into currently non-endemic populations), the overall prevalence of LF in Africa will increase only moderately from the 2000 baseline prevalence of 7.85% to between 9.75–9.85% in 2050.

## Discussion

It is important to note from the outset that our study was primarily concerned with developing a robust analytical method for predicting and mapping LF prevalence distribution and potential future change in Africa in terms of data availability, biological plausibility and model parsimony. However, despite this main objective, the methodology employed in this analysis, viz. using a novel systematically staged ecological covariate selection procedure coupled with selection of a multivariate spatial regression model containing a subset of low-correlated environmental/climatic variables that gave a good fit to the data as well as prediction of observed infection prevalence in test locations [66], has also allowed us to provide improved information regarding the likely environmental determinants of LF prevalence distribution in Africa. Thus, even though the results indicated that spatial structure in the data *per se* played the major part in the Bayesian spatial model’s ability to predict LF prevalence correctly (**Table 2**), six variables belonging to three major environmental themes (viz. climate - mean annual temperature, mean minimum temperature, and mean precipitation in the wettest month; landscape - altitude and NDVI; and population density) are shown to be associated with LF prevalence distribution in Africa. Our use of a staged variable selection process to pre-select a list of predictors could be viewed as setting up the analysis for success [71], and hence could have led to the spurious selection of these covariates. However, using a reduced spatial model containing only the “important” population density variable, did not lead to a marked improvement in model fit nor improve the predictive performance of the full model. This indicates that our pre-selection process did not significantly affect the validity of the inclusion of the fuller set of six variables in our final model. Indeed, the inclusion of these covariates to the Bayesian model incorporating spatial structure only produced a near 90% reduction in DIC values (**Table 2**), indicating that both spatial structure and all six selected covariates were required for describing the observed LF prevalence spatial distribution in Africa.

Several other features of our methodology and the variables selected for use in the final multivariate Bayesian spatial model require to be noted. The first is the need to address the problem of correlation among predictors suitably when developing multivariate regression models [66]. The staged process of variable elimination employed here to produce a candidate list of low or little-correlated predictions from among a larger set of biologically plausible environmental variables as a first stage in our spatial analysis proved to be practical solution to this problem. However, although this method may address the problem of multi-collinearity, it is important to note that excluding variables on the basis of low univariate correlation with the response may obscure the fact that their predictive power may be quite different in the presence of other interacting variables [66]. It is also possible that while these variables may show weak associations individually with a response, they nonetheless may interact together to causally produce the outcome of interest, especially in the case of complex outcomes [72]–[73]. In this study, however, inclusion of all initially evaluated ten variables into the GLSM model did not improve the fit of our final model (data not shown), confirming the relative importance of the selected predictors. Nonetheless, we indicate that further work is required to guide variable selection within a spatial framework particularly in multilevel, multicomponent systems such as parasitic-climate/environment applications, if we are to understand and manage such complex associations more accurately.

The spatial geostatistical modelling framework employed here distinguishes the correlation among observations that can attributed to the spatial proximity of data locations - perhaps generated by the transmission process governed by the flight range of LF mosquito vectors [3] - and that which can be explained by larger-scale first-order spatial correlation explained by common exposures to environmental factors. While the model is thus important for avoiding overestimation of the explanatory power of covariates (compared to a non-spatial model), another significant output of the approach is that it also allows for a better estimation and treatment of residual or unmeasured spatial variation remaining in the model [18]. For example, in the present study, such a formulation can account for the measurement error introduced by the use of prevalence data collated from different studies, each using slightly different sampling and testing methods. Such residual spatial error may also suggest that micro-scale factors, such as those related to poverty, capacity of health facilities, ongoing interventions, and other environmental factors, associated with each location may additionally influence the spatial distribution of observed LF infection. Estimation of the magnitude of such error as afforded by geostatistical analytical frameworks, such as the present model, is thus important as it not only provides a basis for determining the significance of micro-spatial effects but also for supporting further investigations aimed at identifying these additional factors.

Apart from permitting simultaneous environmental risk assessment and modelling of spatial dependence and prediction, the Bayesian modelling framework used here is also important because it allows the quantification of uncertainty in map predictions. Our results arising from such error quantification illustrated via the error map in **Figure 4b** highlight that areas that are more densely sampled for LF infection in Africa (primarily along the west and east Africa coasts and among the Ethiopian highlands) have lower errors associated with their predictions compared to areas in central and south western regions of the continent. The creation and use of such error maps are important in disease control as they indicate areas where additional surveys are required. In the present instance, we indicate that surveys for LF infection prevalence are needed in the above and other areas with large standard errors shown on the map (**Figure 4b**) if we are to develop more accurate maps of LF distribution and prevalence for this continent.

Our best-fit model, overall, indicates that several environmental factors may be associated concurrently with LF infection distribution in Africa. These results have also provided new information regarding the functional forms of their association with LF prevalence, with all the variables selected in our best-fit model – altitude, NDVI, population density, precipitation in the wettest month, mean annual temperature and mean minimum temperature – exhibiting nonlinear quadratic forms for their effects on infection (**Figure 3**). On a technical note, this work has underscored the value of using multivariate models compared to univariate analysis for reliably estimating the function forms of ecological predictors driving disease that are biologically and epidemiological interpretable. Thus, the coefficients for altitude from fitting of the multivariate GLSM imply a strongly negative correlation with LF prevalence. This is consistent with studies that have investigated LF prevalence in relation to altitude [74]–[75] where it is suggested that at higher altitudes, the corresponding lower temperatures (owning to the lapse rate, in which temperature is negatively associated with increasing altitude), would reduce LF transmission due to negative effects on mosquito survival rate and the rate at which the parasite develops within the vector [53], [74]. By contrast, the form of the relationship estimated in the corresponding univariate analysis suggests that altitude may have a more complex non-linear effect on LF prevalence with both an initial positive followed by a negative effect with increasing values (**Figure 2a**), which is clearly more difficult to explain on experimental or ecological grounds. Similarly, the functional form for the mean annual temperature relationship uncovered in the univariate analysis (**Figure 2e**), suggests that LF prevalence may increase non-linearly and non-monotonically with mean annual temperature, whereas in the multivariate case, the relationship is shown to be a monotonic nonlinear one (**Figure 3e**). The latter function is more biological and ecologically plausible given that experimental findings suggest that larval development and mosquito survivorship are maximised at around 22–30°C as [53], [76]. The failure of our functional form to imitate the convexity implied by the experimental results exactly could indicate that mean annual temperature might either not be a useful predictor of LF community prevalence or that our data range is insufficient to conclusively quantify the relationship (given that the upper limit of this variable is restricted at 30 degrees in our study (**Figure 2e**)). The contrast between univariate versus multivariate model estimations of ecological function forms is perhaps most apparent for the NDVI variable (Figure 2b versus **Figure 3b**), which when quantified using a univariate model shows a puzzling decreasing effect on prevalence with increasing values. While this effect may reflect a regulatory effect of forested ecosystems in moderating disease transmission [77], such ecosystem effects might be better captured by the non-linear increase and decrease function shown by the NDVI-prevalence function quantified using the multivariate model. Thus, the multivariate form (**Figure 3b**) suggests that LF prevalence would be highest for NDVI values between 130 and 175, which correspond to shrub or grasslands, or forests, whereas below 130, which refers to barren areas of rock or sand, prevalence, expectedly, is predicted or shown to be low. By contrast, the function indicates that at the highest values of NDVI, prevalence would begin to decrease possibly reflecting the anticipated regulatory effects of forested areas acting to decrease parasite transmission via reductions in vector populations and/or human-vector contacts [77].

Similar complex associations with prevalence are also observed for precipitation values. The association with precipitation levels in the wettest month shows that LF prevalence may increase with precipitation up to a limit of around 1200 mm per month for both models, and above this value, begin to decline. A positive association between LF prevalence and precipitation might be expected because LF vectors require a certain amount of precipitation so that suitable sites to lay eggs are formed. However, our result is the first to support and provide a functional form for the suggestion that when there are heavy downpours these laying sites can get washed away [56], leading to lower overall transmission of infection in areas that experience the highest amounts of precipitation.

In contrast to the purely environmental/climate variables investigated, LF prevalence was found to be associated strongly only with human population density in this study (**Table 2**). While the uncovering of an association between prevalence and host population density is unsurprising given that human to vector density ratios are important drivers of the transmission of vector-borne infections [58], it is clear again that the form estimated by the univariate logistic regression model showing that the association may be negative for LF in Africa may be misleading (**Figure 2c** **)**, as a positive association as predicted by the multivariate GLSM is likely to be the more expected response on theoretical grounds [58]. The statistical evidence for a stronger influence for this variable in comparison with environmental variables *per se* (**Table 2**), however, suggests that this factor may play the biggest role in determining LF prevalence in a location; *ie.* that given a suitable environment for transmission as defined by climatic variables, variations in population density, and more importantly human activities, would play the major role in underlying LF transmission intensity and prevalence levels. This conclusion, and the finding above for NDVI, support suggestions that gaining a better understanding of the association between the environment and LF, and indeed other vector-borne infections, requires a more detailed examination of other non-climatic variables that may confound such relationships and therefore underlie real world parasite transmission, including in particular the socio-ecological dimensions of parasite transmission and the effects of land use or habitat alteration by humans [78].

The multivariate Bayesian GLSM model developed here indicates that LF prevalence distribution in Africa may be highly spatially variable. The map depicted in **Figure 4a** indicates that LF prevalence is high in West Africa, West Central region, along the coast of East Africa, and Sudan, with significantly lower infection prevalence predicted for central Africa and in the northern and southern portions of the continent, except for Egypt. The highest LF prevalence is shown to occur in West Africa, with prevalences higher >23% predicted for Guinea, Guinea-Bissau, Burkina Faso, Senegal and Sierra Leone, while moderately high prevalences are predicted for other West African countries and for the endemic countries of East Africa (**Table 4**). The model further predicts that significant LF infection prevalence (ranging from 6.1% to 12.3%) may occur for several countries, such as Democratic Republic of Congo, Central African Republic and Angola, for which no survey data were available to us for analysis. Similarly, although we have masked the results, the model also predicted infection to occur in several known non-endemic countries, such as South Africa and the other northern Africa countries. While this might indicate the potential for infection in these regions given the covariate distribution in these locations, it is clear that the large errors also predicted for these regions (**Figure 4b**) indicate either absence (as supported by documentation of lack of infection in the latter two regions) or a need to conduct new community surveys in those areas (such as the Democratic Republic of Congo, Central African Republic and Angola) without such clear documentation.

Although the errors behind the generation of the map produced in this work need to be borne in mind, the continuous Africa LF prevalence risk map when used in conjunction with a continuous population density map has allowed a more reliable calculation of the number of people infected with LF in Africa. In total, the overlaying of these maps indicate that some 61.55 million people may have been infected with LF in Africa prior to the advent of large-scale intervention programs, implying that mean LF prevalence was around 7.8% for this continent given a 2000 population of 783.5 million. The last attempt to estimate the burden of LF in Africa [35] estimated that approximately 40 million people were infected. The 1990 population used in their estimate was 726 million, which gave a mean LF prevalence of 5.5% in Africa at that time. However, that figure was based on the assumption of homogenous distribution (unlike the capture of within-country variation by the present continuous map) of prevalence across individual countries, and the disregarding of countries for which no data were available. Thus, the higher LF burden estimated in this study could be a reflection of a better treatment of within country heterogeneities in both infection and population density distributions, and/or the impact of higher overall current population size. Among individual counties, we indicate that those with the largest infected populations in 2000 are found to be in Nigeria (11.01 million), followed by Sudan (7.04 million) and Tanzania (5.40 million), with sizeable infected populations also occurring in Burkina Faso, Uganda, Zaire and Senegal (all close to or >2.5 million) (**Table 4**). This heterogeneity in country-level prevalences clearly imply that efforts to achieve the elimination of LF in Africa will vary markedly between countries, with perhaps more intensive efforts required in the case of those countries exhibiting the highest baseline prevalences [79].

The first result of import from our work on future prevalence change relates to the widely accepted notion that the main effect of climate change on the extent and the severity of vector-borne diseases is to shift rather than increase the geographical range of the diseases [55]. Our findings do not fully agree with this assertion as we predict both changes in severity (**Figure 5**) across Africa, particularly in West Africa, and a potential range shift to the north and south of the continent. Our future climate predictions, however, assume that the biological relationships governing transmission are unchanged between now and 2050. Thus, adaptation by *Wuchereria bancrofti* or the different mosquito vectors to climate change are not taken into account in these first estimates [80], although we note, as per Schmalhausen's law [81], that this is likely to be a major problem only at the extremes of the range of variables that may negatively affect transmission (**Figure 3**). Areas of such extreme changes in the future are also relatively small meaning that vector or parasite biological/physiological adaptation to future climatic changes are unlikely to have a large impact on the results presented here. The sensitivity of our results to different climate scenarios was also explored by running the final model using data from two climate models and two climate scenarios. We found only small differences between the different scenarios/models – the A2a climate scenario lead to a slightly higher prevalence and hence estimates of population infected (211m compared with 184m for the B2a scenario), suggesting that the results are robust to the climate model or development scenario used.

It is clear, however, that given the significance of the contribution of population density in our final model, and the fact that between 2000 and 2050, the population is expected to more than double (http://esa.un.org/unpp/), the primary driver of increased LF burden in Africa will be rapid population growth. This conclusion again underscores the importance of considering the role of non-climatic factors that may modify future climatic influences when assessing possible effects of climate change on infectious diseases [82]–[85]. It also provides an indication into the types of adaptations and policies that may be required to counter the effects of climate change on a particular disease. For example, our results imply that development-related strategies to strengthen LF control programs and possibly reduce population growth in the short term can increase the capacity of endemic African countries to cope with the projected increase in LF over the medium to long run [83], [86]. In the same vein, it might be possible to use estimates of pre-intervention, current and future disease prevalences to investigate whether investing in disease control and wider economic development (which would also bring about knock on effects in reducing population growth) or investing in greenhouse gas emission reduction *per se* would be the better choice in mitigating against the impact of future climate change on parasitic infections [83], [85]. This perspective implies that the current global initiative to control LF may also be considered as an adaptation to a major potential health impact of future climate change, a conclusion which provides an additional rationale for its enaction and successful global implementation.

One of the major limitations of studies aimed at identifying, quantifying and predicting the impacts of environmental/climatic risk factors on parasitic diseases clearly lies in the difficulty of locating and matching the temporal and spatial scales of observational versus climate prediction data [87]. This study is no exception. For example, our baseline prevalence prediction map is based on LF survey data collected between 1940 and 2009, while the WorldClim climate predictor data used in model development are based on averages from 1950 to 2000. Although our infection data are thus reasonably conformal to the WorldClim data, it is apparent that the analysis does not consider the possibility of temporal changes occurring in both infection and climate during the above periods. However, given that systematic interventions against LF began only after 2000 in selected countries [88], and that temperature-related changes began to increase above recent natural fluctuation bounds only just before or around the year 2000 in Africa [70], we suggest that such changes are unlikely to significantly bias the present results. The issue of spatial scale, on the other hand, is a more difficult problem to address, as micro-spatial factors below the 12×12 km model resolution, including different age distributions in study locations, proximity to a water source, survey methodological differences, socio-economic effects, and amount of migration, among others, could also have had an impact in influencing the level of variation in LF prevalence observed at the village level in our study. Similarly, the change from the 12 km model development to a coarser 60 km prediction scale implies that the model fitted at the 12 km resolution is also suitable at the coarser scale, an assumption which may or may not be true. These considerations indicate that the present larger scale spatial predictions may provide only an index of the micro-spatial scale heterogeneity underlying the data.

A second limitation of our study is that the models also have not taken into consideration that the spatial dependence structure in the data may well be different in different regions of Africa. Differences in vector species composition which may influence LF transmission are also likely to vary between different regions making taking a stationary modelling approach questionable. Bayesian non-stationary geostatistical models for quantifying parasitic risk have begun to be developed recently [69], [89]–[90], and we expect that the application of such advances will enable development of more reliable LF, and indeed other parasitic disease, maps in the near future.

Nonetheless, this study has provided the first LF prevalence map for a major endemic region developed using Bayesian geostatistical techniques. This approach has allowed us to estimate the prevalence of LF across Africa at both surveyed and unsurveyed locations based on environmental covariates, spatial dependence between data locations, and a better treatment of sampling error. It has also allowed us to identify and analyse the impacts, and functional forms, of key environmental risk factors for LF, which in turn has provided a hint of the complex multidimensional pathways through which these factors may act, possibly with other non-climatic factors, to influence the transmission of this parasitic disease in Africa. We have used the map to estimate the number of people infected with LF in different countries, and make rough predictions of the impact of changes in climate and population growth on the future growth of the disease on the continent in the absence of control. We hope that by identifying the key spatial risk factors for infection, and those areas with high current and future prevalence or with the potential for emergence of LF infection, control programmes will be able to use the present results to make better informed decisions as to where to focus efforts, assess and develop mitigating stategies for the future, and better quantify the magnitude of efforts required to reduce LF prevalence to either zero transmission or below a suitable infection threshold in any given region of Africa [57], [91].

## Supporting Information

Information S1
**Contains two tables and two figures.** Table S1 – Details of studies and data used in the Bayesian geostatistical analysis. Table S2 – Convergence statistics for the mcmc model fit. Figure S1 – Matrix of scatterplots between all pairs of variables. Figure S2– Trace plots of the beta coefficients and the spatial parameters, 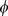 and 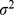.(DOCX)
